# DOC-IDS: A Deep Learning-Based Method for Feature Extraction and Anomaly Detection in Network Traffic

**DOI:** 10.3390/s22124405

**Published:** 2022-06-10

**Authors:** Naoto Yoshimura, Hiroki Kuzuno, Yoshiaki Shiraishi, Masakatu Morii

**Affiliations:** Graduate School of Engineering, Kobe University, Kobe 657-8501, Japan; yoshimura.naoto@gsuite.kobe-u.ac.jp (N.Y.); zenmei@port.kobe-u.ac.jp (Y.S.); mmorii@kobe-u.ac.jp (M.M.)

**Keywords:** deep learning, feature extraction, anomaly detection, convolutional neural network, autoencoder, intrusion detection

## Abstract

With the growing diversity of cyberattacks in recent years, anomaly-based intrusion detection systems that can detect unknown attacks have attracted significant attention. Furthermore, a wide range of studies on anomaly detection using machine learning and deep learning methods have been conducted. However, many machine learning and deep learning-based methods require significant effort to design the detection feature values, extract the feature values from network packets, and acquire the labeled data used for model training. To solve the aforementioned problems, this paper proposes a new model called DOC-IDS, which is an intrusion detection system based on Perera’s deep one-class classification. The DOC-IDS, which comprises a pair of one-dimensional convolutional neural networks and an autoencoder, uses three different loss functions for training. Although, in general, only regular traffic from the computer network subject to detection is used for anomaly detection training, the DOC-IDS also uses multi-class labeled traffic from open datasets for feature extraction. Therefore, by streamlining the classification task on multi-class labeled traffic, we can obtain a feature representation with highly enhanced data discrimination abilities. Simultaneously, we perform variance minimization in the feature space, even on regular traffic, to further improve the model’s ability to discriminate between normal and abnormal traffic. The DOC-IDS is a single deep learning model that can automatically perform feature extraction and anomaly detection. This paper also reports experiments for evaluating the anomaly detection performance of the DOC-IDS. The results suggest that the DOC-IDS offers higher anomaly detection performance while reducing the load resulting from the design and extraction of feature values.

## 1. Introduction

The growth of the Internet in recent years has produced a wide variety of services and improved the convenience of our daily lives. However, this has also resulted in increased numbers of cyberattacks. Intrusion detection systems (IDSs) are one mechanism for detecting such attacks. Such systems can be broadly divided into two categories depending on the detection method used. The first, signature-based IDS, performs detection based on rules that are defined in advance. The second is an anomaly-based IDS that detects abnormal states as anomalies. However, a signature-based IDS cannot detect attacks for which it has no rules, which imposes an extremely large burden on designers by requiring new rules to be added in response to the ever more diverse range of new cyberattacks. Consequently, anomaly-based IDSs that can detect unknown cyberattacks, particularly methods that use machine learning (ML) and deep learning (DL), have attracted significant attention and are now being widely researched [1,2,3].

Autoencoders, which are a core technology among the anomaly detection models that use DL, generally have smaller intermediate layers than their same-sized input and output layers. Because autoencoders are normally trained to reconstruct the input, the input data are compressed into lower dimensionality by the intermediate layer. Hinton et al. [4] described steps up to the intermediate layer as a non-linear generalization of principal component analysis (PCA). When an autoencoder is trained to reconstruct the training data, it experiences difficulty when encountering unfamiliar data that were not present during training. In the security field, attempts have been made to detect such attacks as anomalies by using autoencoders [5,6,7,8]. Furthermore, there are reports of attempts to use convolutional neural networks (CNNs) for intrusion detection, which are used primarily in the image recognition field [9,10,11]. Specifically, CNNs have been proposed as a method for utilizing the relationships between data by learning the features that determine data shapes, arranging and visualizing data in two dimensions, and transforming packet byte arrays into integers.

However, many of these methods are difficult to implement for various reasons. For example, it is necessary to make the feature values used in detection capable of discriminating between normal and abnormal traffic based on network packets, requiring complicated network packet processing during the extraction of the designed feature values. Furthermore, when a supervised learning method is used, labeled data must be obtained and/or created. These difficulties can impose obstacles in the research and practical application of anomaly-based IDSs. Accordingly, our study proposes an anomaly detection method that can perform automatic feature extraction without requiring specially labeled data for each use case.

The DOC-IDS proposed herein is a feature extraction and anomaly detection method that uses a two-input DL model and employs a deep one-class classification (DOC) [12] feature extraction method for one-class classification, which is normally used in the computer vision field, to extract features from network packets. The DOC-IDS consists of a pair of identical one-dimensional (1D) CNNs, one of which is connected to an autoencoder via the intermediate layer. In operation, the DOC-IDS uses these 1D CNNs to extract features that reveal the relationship between bytes in traffic data. During training, an existing labeled multi-class dataset is input in addition to the single class normal traffic. Two loss types are used to improve the discriminative ability among the data, whereas another loss type is used for anomaly detection. The model trained in this manner acquires feature representations with a highly enhanced ability to discriminate between normal and abnormal traffic that did not appear in the training process and is also able to perform anomaly detection. The results of the evaluation experiments using multiple datasets showed that the DOC-IDS has a higher detection accuracy than existing methods, it is particularly effective for detecting communication with command and control (C2) servers, and its processing speed delivers sufficient performance for practical applications.

The contributions of this study can be summarized as follows:

- We apply a feature extraction method for one-class classification, which has high anomaly detection performance in the computer vision field, to traffic data. We then demonstrate a method of obtaining feature representations with a highly enhanced ability to discriminate between normal and abnormal traffic. Furthermore, we reduce the burden of designing and extracting feature values.
- We have simultaneously trained the CNN for feature extraction and the autoencoder for anomaly detection by implementing those processes using a single DL model.
- We show through evaluation experiments that our DOC-IDS method can detect anomalous traffic with high accuracy levels, particularly when handling communications with C2 servers.

The remainder of this paper is organized as follows. Section 2 introduces the related work. Section 3 introduces closely related research, and Section 4 presents the architecture, training method, and anomaly detection method for the proposed DOC-IDS. Section 5 describes the datasets used in the experiments, and Section 6 and Section 7 describe two experiments conducted using the datasets. Finally, Section 8 summarizes the study.

## 2. Related Works

With the growing diversity of cyberattacks in recent years, there is the new burden of creating rules for signature-based IDSs that cannot detect attacks for which they have not learned the correct rules. Research is now focusing on anomaly-based IDSs that can detect unknown attacks using ML and DL [1,2,3].

Numerous methods that use feature values extracted from flows provided by open datasets have been proposed, such as NSL-KDD [13] and CIC-IDS2017 [14]. For example, Zavrak et al. [7] trained an autoencoder, variational autoencoder (VAE), and one-class support vector machine (SVM) using normal traffic flow data contained in the CIC-IDS2017 dataset to perform abnormal traffic detection. Separately, Khan et al. [15] proposed a hybrid convolutional recurrent neural network intrusion detection system (HCRNNIDS) that uses a convolutional recurrent neural network (CRNN) for the flow data contained in the CSE-CIC-IDS2018 dataset. In the HCRNNIDS, feature value engineering is performed on the flow data, and a classifier is trained using flow data labels. Hence, the HCRNNIDS can extract spatial and temporal features using a convolutional layer combined with a recurrent layer. Su et al. [16] proposed the BAT-MC detection model that combined bidirectional long short-term memory with an NSL-KDD convolutional layer. In that study, the authors converted the category variables among the NSL-KDD to a one-hot representation and used the obtained numerical data converted to an image as an input. BAT-MC training was performed by classifying the NSL-KDD labels.

Some existing studies using flows have focused on feature selection. For example, Gül et al. [17] proposed a feature selection algorithm for the NSL-KDD that adopted an attribute evaluator to evaluate each feature and a search method to find feature combinations. The proposed algorithm achieved detection with less execution time, which was almost the same as using all the features. In [18], Alani et al. performed preprocessing such as the binarization of classes into malicious and benign, balancing of data, removal of missing values, and encoding of categorical variables. For feature selection, they proposed a method of classification that employed a random forest and repeated the process of removing features with low contribution rates, starting with 48 features after preprocessing and repeating until five features were selected. Gharaee et al. [19] proposed a feature selection method using a genetic algorithm (GA), in which features obtained by GA were used to train an SVM and classify communications until the maximum number of iterations was reached or the termination criteria were met. They also proposed a fitness value for the GA that multiplies the true positive rate (TPR), false positive rate (FPR), and the number of features by their respective weights.

In methods that use the flow data contained in these datasets, the features are extracted in advance so we can focus on the detection model design. Although there are also many methods that perform feature value engineering to select only the most useful feature values from those provided [20], there have been no studies showing that feature value extraction processing needs to be implemented during actual operations and that labeling is required for detecting attacks when using supervised learning.

Methods that do not use the flow data provided by open datasets have also been proposed to extract features from network packets. For example, Mirsky et al. [5] proposed the Kitsune anomaly detection framework that uses an ensemble of autoencoders. This method also tallies the statistical quantities from the network packets using an incremental method, and it performs feature value extraction based on the obtained statistical quantities. Specifically, the feature values are allocated to the autoencoder ensemble, and anomaly detection is performed by incorporating the reconstruction error for each autoencoder.

Yu et al. [8] extracted features useful for anomaly detection using dilated convolutional autoencoders (DCAEs). The authors accomplished this by extracting information from the header information and network packet payloads and then training the DCAEs by inputting the obtained information arranged in two dimensions. The output from the intermediate layers of the DCAEs provides the feature representation of the traffic data, and a communication classifier is obtained by connecting a fully connected layer to the intermediate layer and then performing fine tuning using labeled data.

Among the methods for extracting features from network packets, some proposals are related to significantly reducing packet processing. For example, the D-PACK method proposed by Hwang et al. [6], which is similar to our proposed method, implements feature extraction from raw packets and abnormal traffic detection using a combination of a CNN and autoencoder. In the D-PACK training process, a set of normally labeled traffic is input, and the CNN classification and autoencoder reconstruction errors are used.

However, even in methods that perform feature extraction from packets, feature value designs, complicated packet processing, and labeled data for supervised learning are required, all of which can be obstacles to the research and practical application of anomaly-based IDSs. Accordingly, when these problems are addressed, the amount of labor involved in the research and practical application of anomaly-based IDSs can be reduced. Thus, programmers can focus on more important problems, such as anomaly detection model design.

With these points, the present study proposes the DOC-IDS method as a feature extraction and anomaly detection method using a two-input DL model. Specifically, the DOC-IDS employs the DOC [12] feature extraction method, commonly employed in the computer vision field, to acquire feature values using a highly enhanced ability to discriminate between normal and abnormal traffic. Although a labeled multi-class dataset is required to improve discriminative ability during DOC-IDS training, DOC-IDS uses existing data from open datasets, which implies that it does not require labeling of the detected network traffic. The primary advantage of this method is that it resolves the problems that have hindered the research and practical application of anomaly-based IDSs in existing studies [5,6,7,8,9,10,11,15,16] that are related to the burden of designing and extracting feature values and creating labels. Furthermore, the DOC-IDS model can simultaneously train feature extraction and anomaly detection networks using a single DL model.

## 3. Learning Deep Features for One-Class Classification

This section describes the one-class classification image feature extraction method developed by Perera et al. [12] that is employed in our proposed method. Because data that contain anomalies and novelties for use in methods aimed at detecting such characteristics are difficult to obtain, the general approach is to use one-class classifications that perform model training using normal data and then detect anomalous and novel characteristics as outliers. However, in the computer vision field, Perera et al. proposed DOC as a method for extracting useful features for one-class classification. In this method, which uses labeled multi-class data for domains other than the one-class classification target, the authors perform general anomaly and novelty detection training, during which they refer to images that do not fit the given single class as belonging to an alien class. To accomplish this, the DOC uses two different loss types to increase its ability to discriminate between alien and pre-assigned single-class images. The DOC network trained using these loss functions comprises a pair of identical CNNs (reference and secondary networks) that share weights, and each CNN is divided into subnetwork *g* (which performs feature extraction) and subnetwork $h_{c}$ (which performs classification). DOC is explained in more detail below.

### 3.1. Reference Network

The role of the reference network is to maintain the ability to discriminate between data. Therefore, a labeled multi-class reference dataset is used as the input, rather than a single-class image set that normally provides the one-class classification target. A descriptiveness loss calculation ($l_{D}$) is performed to increase a network’s discriminative ability. Perera et al. aimed to maximize the distance between image classes using cross-entropy loss, which is expressed as $l_{D}$ in Equation (1). Note that *n* is the mini-batch size, $y_{i}$ and *k* are, respectively, the output and size of $h_{c}$, and $t_{i}$ is the training dataset.

(1) $$l_{D}=-\frac{1}{n}\sum_{i=1}^{n}\sum_{j=1}^{k}t_{ij}log\left(y_{ij}\right).$$

### 3.2. Secondary Network

The role of the secondary network is to compactly distribute the target data of the one-class classification in the feature space. Therefore, a single-class dataset, which is the one-class classification target, is used as the input, and the compactness loss ($l_{C}$) that represents the distribution of the $h_{c}$ outputs is calculated as the loss. Perera defined $l_{C}$, as expressed in Equation (2) and calculated $l_{C}$ for each mini-batch. Note that $\sigma_{i}^{2}$ represents the $h_{C}$ output variance.

(2) $$l_{c}=\frac{1}{nk}\sum_{i=1}^{n}\frac{n^{2}\sigma_{i}^{2}}{{\left(n-1\right)}^{2}}.$$

### 3.3. Training

When training starts, the CNN is initialized with the weights of the trained model, after which the weights (except for the last four layers) are fixed. Furthermore, during training, the reference and target datasets are provided as inputs to the reference and secondary networks, respectively. The overall model is trained using the combined loss of the two obtained loss types ($l_{D}$ and $l_{C}$) in Equation (3). Perera et al. set the coefficient λ (representing the importance of $l_{C}$) to 0.1.

(3) $$loss=l_{D}+\lambda l_{C}.$$

### 3.4. Feature Extraction

When the model training is completed, the output of *g* is obtained as a feature value for one-class classification. This output is expected to represent the differences in data between different classes owing to $l_{D}$, and the single-class data, which are the target of the one-class classification, are expected to be compactly distributed by $l_{C}$.

### 3.5. Application for Anomaly Detection

Perera et al. [12] proposed a method that uses a one-class classifier trained with the subnetwork *g* output, which is then trained against normal data, to use DOC for anomaly detection. One-class classifiers include one-class SVMs [21], support vector data description (SVDD) [22], and k-nearest neighbor classification. The results of their anomaly detection experiments conducted on an image dataset revealed high anomaly detection accuracy levels in a variety of cases. Our DOC-IDS applies the DOC method to network packets and implements the processes from feature extraction to anomaly detection using a single DL model that connect an autoencoder to a CNN.

## 4. DOC-IDS: Deep Learning Model for Feature Extraction and Anomaly Detection

This section describes the proposed DOC-IDS method, which implements automatic feature extraction and anomaly detection from network packets using a single DL model.

### 4.1. Overview

**Figure 1:**
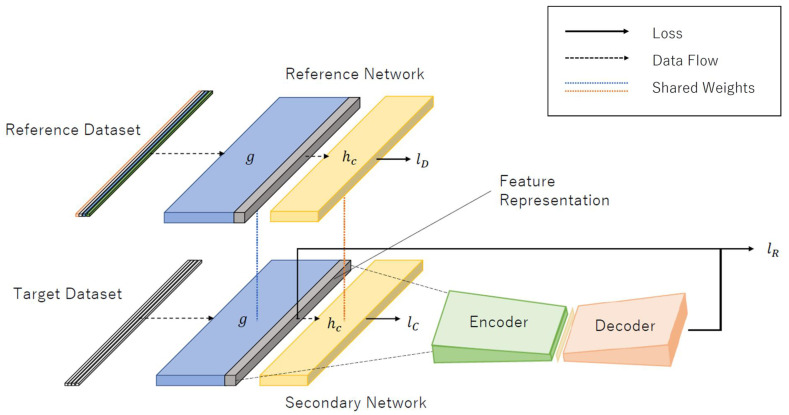
Architecture of DOC-IDS. The reference and target datasets used as input are multi-class and single-class datasets, respectively. The labels *g* and $h_{C}$ refer to the CNN subnetworks, which are responsible for feature extraction and classification, respectively. The labels $l_{D}$, $l_{C}$, and $l_{R}$ refer to the losses computed for each output.

As explained previously, the DOC-IDS is a method that extracts features and detects anomalies from network packets using a single DL model. To accomplish this, the DOC-IDS first performs a flow sampling. Here, the flow is defined as communication divided into five tuples, each consisting of the source/destination internet protocol (IP) address, port number, and transport layer protocol number. Training is then performed using the sampled values as the input. The DOC-IDS comprises 1D CNN and autoencoder components for feature extraction and anomaly detection, respectively (Figure 1). The 1D CNN was used for feature extraction because it can understand the relationships between each byte in the traffic data. For training, a target dataset containing normal traffic from the target computer network and a reference dataset containing labeled multi-class traffic from an open dataset were used. At this time, three loss types are used for training, each of which has the purpose of enhancing the network’s ability to discriminate between data, minimizing the variance of normal traffic in feature space, and minimizing the autoencoder reconstruction error. Anomaly detection is performed in the model using the autoencoder reconstruction error.

### 4.2. Architecture

The DOC-IDS architecture comprises reference and secondary networks. The reference network dataset, which is a labeled multi-class version, uses a domain different from the detection target as the input. In contrast, the secondary network dataset uses one-class data from the target computer network as the input. The reference and secondary networks have identical CNNs that share weights, and these CNNs can be thought of as consisting of subnetworks *g* and $h_{c}$. In the secondary network, in addition to the CNN, an autoencoder for anomaly detection is connected via subnetwork *g*. Table 1 lists the layers in each component. Various DOC-IDS components are explained in detail below. In determining the parameters of the DOC-IDS, the output size was adjusted and tuned based on the model proposed by Hwang et al. [6].

#### 4.2.1. Reference Network

The reference network was trained to increase its ability to detect anomalies in a target dataset. To accomplish this, a reference dataset, which is an existing labeled multi-class dataset (such as an open dataset), was used as the input for calculating $l_{D}$ to discriminate the differences between the various classes. This allows for a feature representation for discriminating between traffic types to be obtained from the model *g* output that is trained for the classification task. For the reference network loss, the cross-entropy loss in Equation (1) is calculated from the $h_{c}$ output using the method described by Perera et al.

#### 4.2.2. Secondary Network

The secondary network CNN is identical to the one used in the reference network and is connected to an autoencoder from subnetwork *g*. A target dataset containing single-class data from the computer network (on which anomaly detection will be performed) was used for training the secondary network. During training, two loss function types were calculated to minimize both the output variance of $h_{c}$ on the target dataset and autoencoder reconstruction errors. The compactness loss ($l_{C}$) in Equation (2) proposed by Perera et al. was used to minimize the variance. This loss is expected to result in a feature representation with a highly enhanced ability to discriminate between normal and abnormal traffic. Note that $l_{C}$ was calculated for each mini-batch used in the training process. The reconstruction loss ($l_{R}$) for minimizing the autoencoder reconstruction error used the mean squared error (MSE) of the output $g_{i}$ of subnetwork *g*, which is introduced as follows:

(4) $$l_{R}=\frac{1}{no}\sum_{i=1}^{n}\sum_{j=1}^{o}{\left(y_{ij}-g_{ij}\right)}^{2}.$$

### 4.3. Sampling Network Flow

The method described by Hwang et al. [6] was used for the flow sampling. Their study also proposed an anomaly detection method that uses only *l* bytes from each of the first *n* packets from the flow aggregated by five-tuple values, which are defined based on the source IP address, destination IP address, source port number, destination port number, and the transport layer protocol number. This method can not only significantly reduce the amount of data to be processed but also enable earlier anomaly detection. Although the authors recommended n=2 and l=80 in their paper, our research adopted n=4 amd l=80 to consider the payload following the three-way handshake in transmission Control Protocol (TCP) communications.

During flow sampling, the packets that are represented by a byte array were converted to integer values of 0–255 bytes at a time. Any portion in excess of length *l* was discarded, and zero-padding was performed on packets that are shorter than length *l*. Furthermore, to prevent the feature extraction and anomaly detection functions from focusing on the sender as discriminative information, an anonymization processing was performed to change the IP and media access control (MAC) addresses in the training data to random values.

### 4.4. Training

The loss in Equation (5), which is the combination of the three loss types $l_{D}$, $l_{C}$, and $l_{R}$, was used for training a model in DOC-IDS. The coefficients $\lambda_{D}$, $\lambda_{C}$, and $\lambda_{R}$ are positive constants that represent the importance of each loss in the learning process. Our study used $\lambda_{D}=1$, $\lambda_{C}=0.1$, and $\lambda_{R}=10$. Refer to Appendix A for details on the effect of changing $\lambda_{R}$ on the accuracy levels. Furthermore, the stochastic gradient descent (SGD) was used as the optimization algorithm, the learning rate was set to $5\times 10^{-5}$, and the weight decay is set to $5\times 10^{-5}$. The weights of subnetworks *g* and $h_{c}$ of the reference and secondary networks were always shared during training.

(5) $$loss=\lambda_{D}l_{D}+\lambda_{C}l_{C}+\lambda_{R}l_{R}.$$

**Figure 2:**
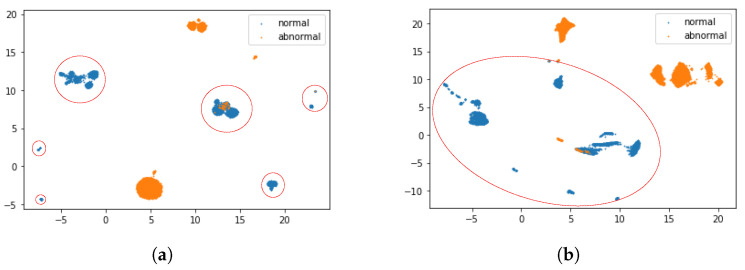
Visualization of data input to the DOC-IDS and feature representation by the DOC-IDS using UMAP. The blue and orange dots indicate normal and abnormal traffic, respectively. The normal traffic is circled by a red line. (**a**) Input to DOC-IDS (320 dimensions). (**b**) Output from subnetwork g (256 dimensions).

Figure 2 shows a visualization of the output of the subnetwork *g*, which is the input to the autoencoder component and the input data to the DOC-IDS using Uniform Manifold Approximation and Projection (UMAP) [23]. As shown in Figure 2, in the features extracted by the DOC-IDS, normal communication is distributed in a relatively small area.

### 4.5. Detection

**Figure 3:**
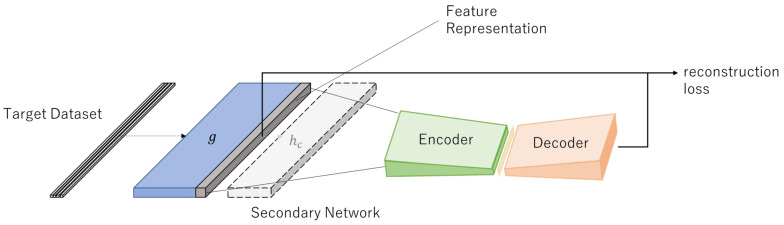
DOC-IDS test mode.

**Figure 4:**
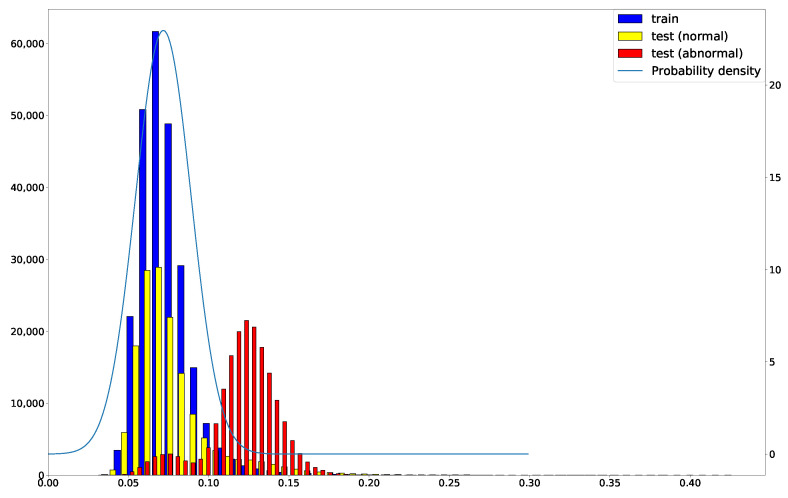
Reconstruction error distribution in DOC-IDS. The blue, yellow and red histograms show the training data error, normal traffic error in the test data, and abnormal traffic error in the test data, respectively. The blue line shows the probability density function for the training data error.

When anomaly detection is performed, the reference network diverges from the trained model (Figure 3). Anomaly detection is performed by using the MSE of the autoencoder reconstruction error as the anomaly score. Figure 4 shows an example of the *g* output reconstruction error of the DOC-IDS.

The threshold value, which is used to determine whether an anomaly exists, was set using the training data reconstruction error (the distribution is represented in blue in Figure 4). First, the mean value μ and standard deviation σ were calculated from the reconstruction error in the training data, after which these values were used to fit the following normal distribution:

(6) $$\frac{1}{\sqrt{2\pi \sigma^{2}}}\exp\left(-\frac{{\left(x-\mu \right)}^{2}}{2\sigma^{2}}\right).$$

When fitting to a normal distribution, the value range of MSE is [0,∞). However, the probability density in the negative region of the approximately fitted normal distribution is small. Therefore, the impact of the approximation is considered negligible.

The 99% point on the lower side of the obtained normal distribution was then used as the threshold value.

## 5. Datasets

This section describes the datasets used for the experiments in Section 6 and Section 7. As explained previously, two datasets were provided for the reference and target datasets (Table 2). For the packet capture (pcap) files that record the traffic data captured from the networks (i.e., raw packets) treated in this study, traffic-type identification must also be performed because there are a very large number of packets. However, labeling individual traffic is difficult, and the labeling accuracy depends on the data creator. Therefore, reference datasets selected for use were divided into separate pcap files for each class of traffic to ensure the datasets have the highest accuracy level.

### 5.1. Reference Dataset

#### 5.1.1. USTC-TFC2016

The first reference dataset used was the USTC-TFC2016 dataset [11] constructed by Wang et al., which contained 10 classes each for normal and malware traffic. The malware traffic in USTC-TFC2016 was collected from a real network environment by the Czech Technical University (CTU) [24] from 2011 to 2015, whereas a network simulation device of Ixia Breaking Point System (IXIA BPS) [25] was used to collect normal traffic. Table 3 lists the data used in training.

#### 5.1.2. ISCX-VPN-Tor

The ISCX-VPN-Tor dataset was constructed by combining two datasets. The first was the ISCXVPN2016 dataset [26], which contains a virtual private network (VPN) and non-VPN traffic. This dataset includes traffic from multiple applications such as Skype and Facebook to handle multiple traffic types, such as voice over Internet protocol (VoIP) and peer-to-peer (P2P). Wireshark [27] was used to capture packets, and OpenVPN [28] was used to connect a VPN service to a VPN provider. Furthermore, a service provider was used to generate a secured file transfer protocol and file transfer protocol over secure sockets layer/transport layer security traffic, and FileZilla [29] was used to make the connections.

The second dataset is ISCXTor2016 [30]. This dataset contains Tor and non-Tor traffic, with traffic from multiple applications for multiple traffic types captured by Wireshark in the same manner as the ISCXVPN2016 dataset. The Whonix [31] operating system (OS), which anonymizes traffic using Tor, was used to collect Tor traffic in the ISCXTor2016 dataset. Whonix is made up of two virtual machines called Gateway and Workstation, within which the Workstation communicates with the Internet via the Gateway. In the ISCXTor2016 dataset, the Gateway and Workstation traffic were captured as Tor and non-Tor traffic, respectively. In our experiment, the pcap file contained in the dataset was allocated to classes based on their traffic types for use as a reference dataset. Table 4 presents the training data used in this study.

### 5.2. Target Dataset

#### 5.2.1. BOS 2018

The BOS 2018 dataset, which was extracted from the Anti Malware Engineering Workshop (MWS) Dataset 2018 [32], was used as the first target dataset. The BOS 2018 dataset, which assumes intrusion activities into the internal network of an organization and contains observation data recordings of targeted attacks, is widely used to evaluate intrusion detection models. The communication content was obtained by recording traffic after executing a malware specimen attached to a targeted attack email and then assigning a progress indicator depending on the progress of intrusion activities. A honeypot was used as the environment for observing the dynamic activities of the malware specimen, and the client device on which the malware was executed was able to access the Internet either via a proxy or an other method. BOS2018 is a dataset created by a Japanese organization and was adopted for evaluating the anomaly detection performance of the DOC-IDS for practical use in Japan.

In this experiment, a file containing Progress-2 traffic, which was produced before the C2 server generated traffic, was used for training, and files containing Progress-7 and 8 traffic, which are C2 server communications, were used for testing. The C2 server traffic was labeled as attack traffic. Table 5 lists the data used in this experiment.

#### 5.2.2. CIC-IDS2017

The CIC-IDS2017 dataset [14], which was used as the second target dataset, provided pcap files that captured traffic on weekdays (Monday to Friday), and attack traffic was included in all files, except for Monday. An experimental testbed composed of a victim-network and an attack-network was constructed to collect this traffic. The victim-network comprises a firewall, router, switch, and devices with various OSs. The B-profile system, which generates traffic by profiling the properties of human traffic, hypertext transfer protocol (HTTP), HTTP secure, FTP, secure shell (SSH), and email traffic for 25 users, was proposed and used for the normal traffic generated by this victim-network. The attack-network, which was kept separate from the victim-network, consisted of a router, switch, and devices for executing the attacks. Traffic created by existing tools and attack codes were executed to generate the attack traffic. The CIC-IDS2017 was selected because it covers a wide variety of attacks. CIC-IDS2017 provides pcap files that are divided by day, making it suitable for evaluating the detection speed in Section 7.

In this experiment, the Monday traffic file was used for training, and the detection accuracy was verified using Tuesday–Friday files. Note that Heartbleed and Infiltration were excluded from the attacks because there were insufficient data for labeling. Table 6 lists the data used in the experiment.

## 6. Detection Performance

In this section, we evaluate the anomaly detection performance of the DOC-IDS. The accuracy levels of the autoencoder and 1D convolutional autoencoder when given the same input as the DOC-IDS were also investigated for comparison purposes.

### 6.1. Performance Index

The evaluation indicators used in the experiment are as follows:

The area under the curve (AUC) for the receiver operating characteristic (ROC) and precision–recall (PR) curves were used for evaluation in this experiment. The ROC curve takes the FPR as the horizontal axis and the TPR as the vertical axis, whereas the PR curve takes recall as the horizontal axis and precision as the vertical axis. The anomaly detection performance of the DOC-IDS was based on the threshold value indicated by the precision, recall, and F-measure. Each indicator is given by the following equations, which are based on Table 7:

(7) $$\mathrm{TPR}(\mathrm{Recall})=\frac{\mathrm{TP}}{\mathrm{TP}+\mathrm{FN}}.$$

(8) $$\mathrm{FPR}=\frac{\mathrm{FP}}{\mathrm{FP}+\mathrm{TN}}.$$

(9) $$\mathrm{Precision}=\frac{\mathrm{TP}}{\mathrm{TP}+\mathrm{FP}}.$$

(10) $$F-\mathrm{measure}=\frac{2\cdot \mathrm{Precision}\cdot \mathrm{Recall}}{\mathrm{Precision}+\mathrm{Recall}}.$$

### 6.2. Results

**Figure 5:**
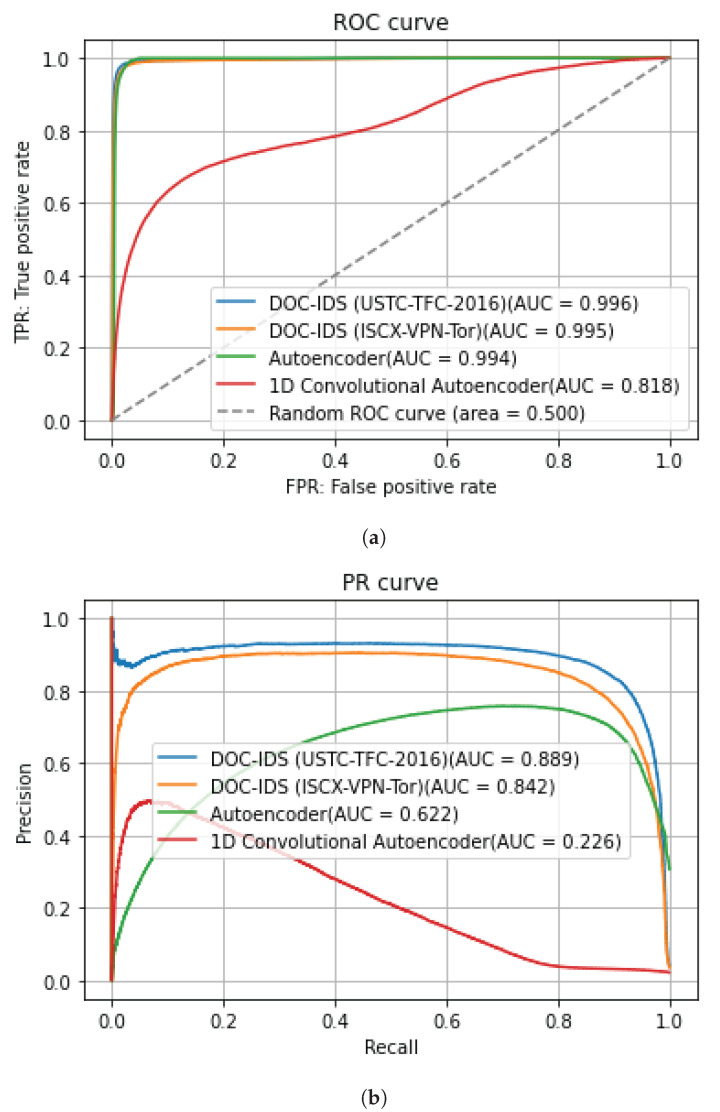
BOS 2018 anomaly detection results for each method. (**a**) ROC curve. (**b**) PR curve.

**Figure 6:**
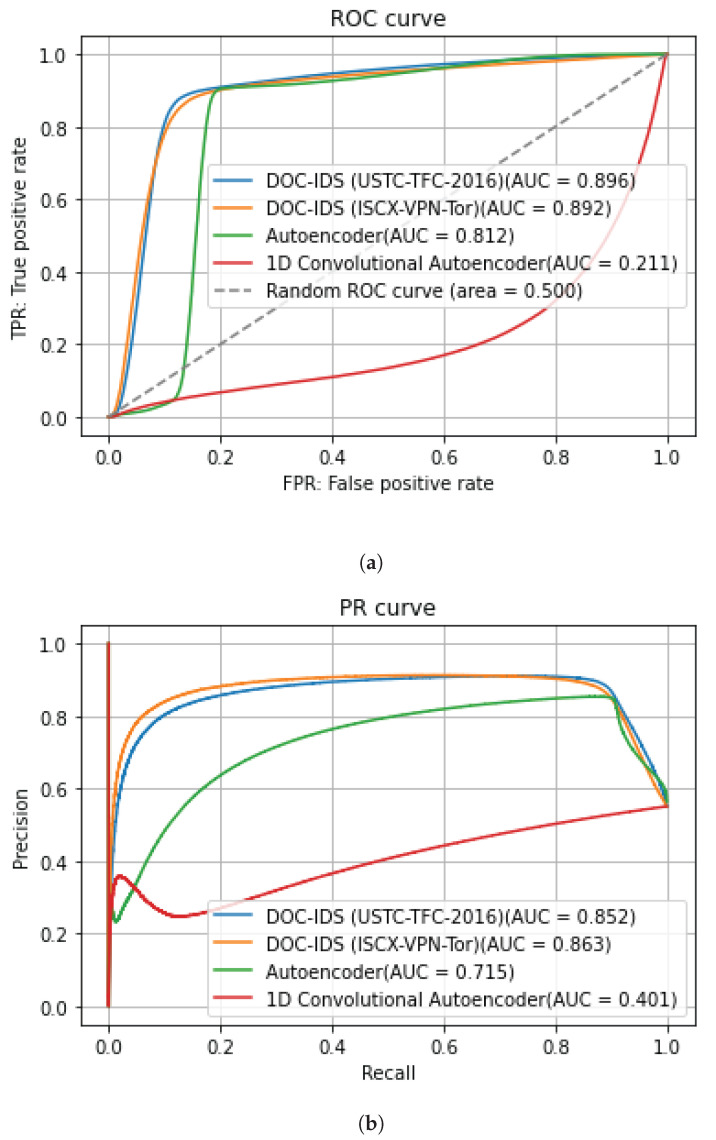
CIC-IDS2017 anomaly detection results for each method. (**a**) ROC curve. (**b**) PR curve.

**Figure 7:**
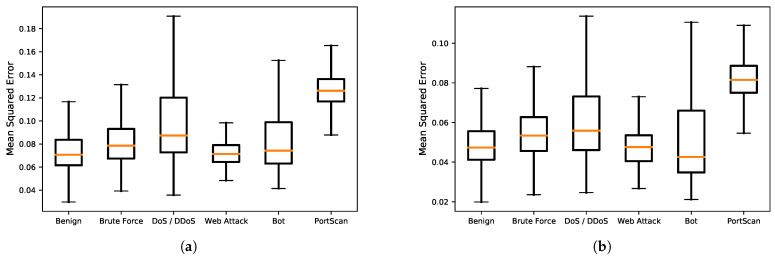
Reconstruction error distribution by attack type. (**a**) Using USTC-TFC-2016 as the reference dataset during training. (**b**) Using CIC-IDS2017 as the reference dataset during training.

Figure 5 and Figure 6 show the AUC for the ROC and PR curves for BOS 2018 and CIC-IDS2017, whereas Table 8 and Table 9 show the detection accuracy levels when the threshold values are used. The figures show that the AUC accuracy for DOC-IDS is higher than that for the other methods for both the ROC and PR curves in the BOS 2018 and CIC-IDS2017 datasets, thereby indicating that it is possible to discriminate between normal and abnormal traffic. In particular, the AUC accuracy significantly exceeds that of the other methods for the BOS 2018 dataset. However, for the CIC-IDS2017 dataset, even though the AUC surpassed the other methods, it clearly had problems detecting some traffic types. Figure 7 shows the reconstruction error distribution for each traffic type. In this figure, there were virtually no brute force or web attack detections in regions with large reconstruction errors that do not contain normal traffic. This indicates that detecting brute force and web attacks is difficult because the differences between reconstruction errors from normal traffic are smaller, implying that it is not possible to set a threshold value to distinguish between them.

### 6.3. Discussion

The results of the anomaly detection performance experiment showed that DOC-IDS detected abnormal traffic with higher accuracy than the comparison methods. Furthermore, these results show particularly high accuracy for the BOS 2018 dataset, and the DOC-IDS appears to have high performance for detecting communications with C2 servers. The superior DOC-IDS feature extraction mechanism contributed to this result. Although the brute force and web attacks included in CIC-IDS2017 were difficult to detect, web attacks could potentially be handled through the combined use of a DOC-IDS and a web application firewall.

A comparison of the characteristics of the DOC-IDS and other methods [5,6,8,15,16] is presented in Table 10. The study being compared is a recent study that used DL and is closely related to this study. The DOC-IDS is, by far, the least burdensome to deploy compared with other methods. In terms of detectable attack types, while attacks are detected in [8,15,16] by classification, the DOC-IDS has relatively few restrictions because it is based on an anomaly detection method, although some attack types (e.g., brute force and web attacks) are difficult to detect.

Regarding the detection of brute force and web attacks, one method to further improve the anomaly detection performance of the DOC-IDS in the future might be to increase the discriminative ability of the feature representation. Hence, self-supervised learning (SSL), which is a method for performing training without using pre-labeled data, can be used to improve DOC-IDS training. This method, which uses labels created mechanically from unlabeled data, has already achieved success in the computer vision field [33], where accuracy levels close to supervised learning have been recorded in ImageNet [34] classification tasks [35]. It is also possible that large amounts of data that do not have labels in the reference dataset, which is currently limited to labeled data, may be useful in the future. Furthermore, a feature representation with an even higher discrimination ability may be obtained by using SSL to improve DOC-IDS training.

## 7. Time Efficiency

Then, the processing performance of the DOC-IDS was evaluated using the CIC-IDS2017 dataset. Table 11 lists the performance of the hardware and software used in the experiments.

In this experiment, the model trained using the Monday traffic file, which does not contain attack traffic, was used to evaluate the time required for anomaly detection. The time measurement was performed for each file contained in CIC-IDS2017, and the time taken for the entire execution, the times taken for both flow sampling, and detection times were investigated.

In terms of implementation, the scapy [36] sniffer method was used to parse the packets. Furthermore, TCP and user datagram protocol (UDP) traffic was processed in parallel to the flow sampling, and the DOC-IDS anomaly detection (divided into five parallel processes) was performed.

### 7.1. Results

Table 12 shows the time taken for the entire execution, whereas Table 13 shows the time taken for detection in the experiments. The experimental results showed that the DOC-IDS processing performance is approximately 5152 packets per second (pps). Table 13 also shows that the majority of the processing time resulted from packet parsing, which depended on the performance of the Scapy library. For flow sampling, the average was approximately 21,964 pps for TCP and 14,435 pps for the UDP. These flow samplings indicated that processing at a maximum of approximately 36,399 pps is possible. Furthermore, because the five above-mentioned detection processes were executed in parallel, the processing could eventually reach 1917 flows per second, and that speed might even be further improved by increasing the degree of parallelism.

### 7.2. Discussion

From the experimental results, we can observe that if we regard the flow sampling processing performance as the bottleneck, the DOC-IDS should be able to process traffic at several tens of megabits per second (Mbps) in an experimental environment, indicating that it can process medium-sized networks. Methods for further speed increases could include using a high-speed parser, using higher-performance hardware, and implementation using a high-performance language, such as C++.

## 8. Conclusions

This paper proposed the DOC-IDS method to reduce the obstacles to the implementation of anomaly-based IDS, which is a method that has been attracting significant attention in recent years. Our method alleviates the difficulties of designing feature values, the complexity of processing in feature value extraction, and the labor required to create labeled data in supervised learning. In our experiments, the DOC-IDS was able to perform processing from feature extraction to anomaly detection without requiring labeling by inputting pre-labeled traffic from an open dataset and the traffic from the target network into the model.

Our experimental results showed that the anomaly detection performance of the DOC-IDS exhibited a maximum AUC for the ROC and PR curves of 0.996 and 0.889, respectively, which surpasses the comparison methods. Furthermore, the processing performance levels are sufficient for practical use. In addition, the DOC-IDS addresses the obstacles in conventional anomaly-based IDS methods using ML and DL by eliminating the need to create specially labeled data or process network packets. Thus, this paper provides interesting implications for future research and practical applications.

## Figures and Tables

**Table 1:** Structural parameters of DOC-IDS.

Network	Layer	Type	Filters/Neurons	Stride	Padding
*g*	1	1D-Conv + Batch Normalization	32 (kernel size = 6)	1	5
*g*	2	Maxpooling	kernel size = 2	2	-
*g*	3	1D-Conv + Batch Normalization	64 (kernel size = 6)	1	5
*g*	4	Maxpooling	kernel size = 2	2	-
*g*	5	Dense + Batch Normalization	1024	-	-
*g*/Autoencoder	6	Dense + Batch Normalization	256	-	-
$h_{c}$	7	Dense	classes	-	-
		(Used only during training)	(the number of classes in reference dataset)		
Autoencoder	7	Dense	128	-	-
Autoencoder	8	Dense	64	-	-
Autoencoder	9	Dense	128	-	-
Autoencoder	10	Dense	256	-	-

**Table 2:** Datasets.

Dataset Type	Dataset
Reference Dataset	USTC-TFC2016
	ISCX-VPN-Tor
Target Dataset	BOS 2018
	CIC-IDS2017

**Table 3:** USTC-TFC2016.

	Class	Number		Class	Number
Normal	BitTorrent	7517	Malware	Cridex	16,385
	FTP	101,037		Geodo	40,945
	Facetime	6000		Htbot	6339
	Gmail	8629		Miuref	13,478
	MySQL	86,089		Neris	33,791
	Outlook	7524		Nsis-ay	6069
	SMB	38,937		Shifu	9631
	Skype	6321		Tinba	8503
	Weibo	39,950		Virut	33,103
	WorldOfWarcraft	7883		Zeus	10,970

**Table 4:** ISCX-VPN-Tor-2016.

Class	Number
VoIP	206,502
Audio-Streaming	2788
Browsing	33,527
Chat	22,248
FTP	82,122
Email	6086
P2P	42,133
Video-Streaming	12,714

**Table 5:** BOS 2018.

Type	Train Data	Test Data
Normal traffic	152,348	659,835
Attack traffic (Progress-7)	-	12,051
Attack traffic (Progress-8)	-	3041

**Table 6:** CIC-IDS2017.

Type	Attack	Train Data	Test Data
Benign	-	249,044	150,618
Brute Force	FTP-Patator	-	2457
	SSH-Patator	-	2905
DoS/DDoS	slowloris	-	3518
	Slowhttptest	-	3610
	Hulk	-	9535
	GoldenEye	-	6592
Web Attack	Brute Force	-	143
	XSS	-	18
	SQL Injection	-	7
Bot		-	1207
Port Scan		-	154,571

**Table 7:** Confusion Matrix.

		Predicted	
		Positive	Negative
Actual	Positive	TP	FN
	Negative	FP	TN

**Table 8:** Detection accuracy on BOS 2018 when using a threshold value.

	Reference Dataset	
	USTC-TFC2016	ISCX-VPN-Tor
Precision	0.773	0.654
Recall	0.939	0.966
F-measure	0.848	0.780

**Table 9:** Detection accuracy on CIC-IDS2017 when using a threshold value.

	Reference Dataset	
	USTC-TFC2016	ISCX-VPN-Tor
Precision	0.911	0.909
Recall	0.756	0.730
F-measure	0.826	0.810

**Table 10:** Comparison of the characteristics of the anomaly detection approach.

Feature	HCRNNIDS [15]	BAT-MC [16]	Yu et al. [8]	Kitsune [5]	D-PACK [6]	DOC-IDS
Packet based			✓	✓	✓	✓
Feature selection is unnecessary		✓		✓	✓	✓
Feature design is unnecessary	✓	✓			✓	✓
Label is unnecessary				✓		✓
Simultaneous feature extraction and detection				✓	✓	✓
Versatility to attack types				✓	✓	✓

**Table 11:** Hardware and software used for processing performance experiments.

OS	Ubuntu 20.04.3 LTS
CPU	Intel(R) Core™ i7-10700 CPU @ 2.90 GHz (16 cores)
Memory	32 GB
Language	Python 3.8.10
Framework	Keras 2.8.0 (Backend: TensorFlow 2.8.0)

**Table 12:** Execution Time.

	Day			
	Tuesday	Wednesday	Thursday	Friday
file size [GB]	10.29	12.50	7.73	8.23
packets	11,551,954	13,788,878	9,322,025	9,997,874
sampling (TCP) [s]	482.87	581.73	393.77	422.77
sampling (UDP) [s]	52.43	51.81	49.27	50.44
detect (Process 1) [s]	109.17	117.96	116.84	125.98
detect (Process 2) [s]	108.63	117.80	116.75	126.10
detect (Process 3) [s]	109.02	117.46	116.74	126.18
detect (Process 4) [s]	108.75	117.47	116.98	126.01
detect (Process 5) [s]	108.84	117.36	117.05	125.78
total [s]	2253.62	2654.35	1798.46	1959.21

**Table 13:** Time Efficiency.

	Day				
	Tuesday	Wednesday	Thursday	Friday	Average
overall [pps]	5125.95	5194.82	5183.34	5103.01	5151.78
sampling (TCP) [pps]	22,180.53	22,249.52	21,683.11	21,741.72	21,963.72
sampling (UDP) [pps]	14,477.24	14,705.39	14,227.92	14,330.46	14,435.25
detect (Process 1) [flows per second]	385.37	383.94	386.95	376.84	-
detect (Process 2) [flows per second]	385.37	383.89	386.70	376.79	-
detect (Process 3) [flows per second]	385.94	384.73	386.60	376.65	-
detect (Process 4) [flows per second]	386.73	384.97	386.47	377.02	-
detect (Process 5) [flows per second]	385.93	384.83	386.28	376.68	-
total of five processes [flows per second]	1929.34	1922.36	1933.00	1884.08	1917.195

## Appendix A

This appendix shows the effect on accuracy for the case in which $\lambda_{R}$, which corresponds to the autoencoder reconstruction error, is changed among the weights for each loss. The values of $\lambda_{D}$ and $\lambda_{C}$ were fixed at 1 and 0.1, respectively, as in Perera et al.

**Table A1:** AUC of ROC Curve.

$\lambda_{R}$	1	5	10	20	50
BOS 2018 (USTC-TFC2016)	0.992	0.996	**0.996**	0.997	0.989
BOS 2018 (ISCX-VPN-Tor)	0.959	0.990	**0.995**	0.986	0.995
CIC-IDS2017 (USTC-TFC2016)	0.866	0.892	**0.896**	0.904	0.901
CIC-IDS2017 (ISCX-VPN-Tor)	0.706	0.903	**0.892**	0.837	0.879

**Table A2:** AUC of PR Curve.

$\lambda_{R}$	1	5	10	20	50
BOS 2018 (USTC-TFC2016)	0.830	0.884	**0.889**	0.911	0.759
BOS 2018 (ISCX-VPN-Tor)	0.448	0.775	**0.842**	0.752	0.829
CIC-IDS2017 (USTC-TFC2016)	0.824	0.827	**0.852**	0.854	0.857
CIC-IDS2017 (ISCX-VPN-Tor)	0.665	0.875	**0.863**	0.791	0.848
